# Fight to survive: Marchantia synthesizes newly identified metabolites in response to wounding

**DOI:** 10.1093/plphys/kiaf066

**Published:** 2025-03-04

**Authors:** Eugenia Pitsili, Aida Maric

**Affiliations:** VIB—Ugent Center for Plant Systems Biology, Department of Plant Biotechnology and Bioinformatics, Ghent University, 9052 Ghent, Belgium; Assistant Features Editor, Plant Physiology, American Society of Plant Biologists; CIBSS-Centre for Integrative Biological Signalling Studies, University of Freiburg, 79104 Freiburg, Germany; Plant Environmental Signalling and Development, Institute of Biology III, University of Freiburg, Schänzlestraße 1, 79104 Freiburg, Germany

Plants sense and adjust to continuously changing environmental cues. Whether these cues are abiotic like temperature and light, or biotic like soil microorganisms, plant responses to all of them are centrally coordinated through phytohormones. The field of phytohormone research has come a long way since the beginning of the last century when “phytohormone” simply meant auxin (Kende and Zeevaart 1997). Besides a group of “classic” hormones—auxin, gibberellin, ethylene, cytokinin, and abscisic acid—the field currently includes an extended group of small-molecule phytohormones that regulate different aspects of plant life and that arose over the course of their evolution (Kende and Zeevaart 1997; Gasperini and Howe 2024).

In terms of signaling pathways, one of the best understood phytohormones is jasmonate (JA). In angiosperms, JA conjugated to isoleucine (JA-Ile) is a major regulator of biotic and abiotic plant stress responses as well as developmental pathways (Chini et al. 2016). It controls diverse processes, from the scent of jasmine flowers to the regulation of complex transcription networks (Chini et al. 2016; Howe et al. 2018). The wide range of responses tightly regulated by JAs is a result of increasing evolutionary complexity and redundancy in its regulatory pathway. While JAs were already present in the earliest land plants and while different plant lineages share JA basic signaling pathway characteristics, the starting molecule that activates the pathway is different for different species (Chini et al. 2023). The lineages that led to *Marchantia polymorpha* and *Arabidopsis thaliana* diverged 450 million years ago. While in bryophyte Marchantia, dinor-12-oxo-phytodienoic acid (dn-OPDA) is the activating JA; in angiosperm Arabidopsis, the JA-Ile conjugate acts as the activator (Fig).

**Figure:**
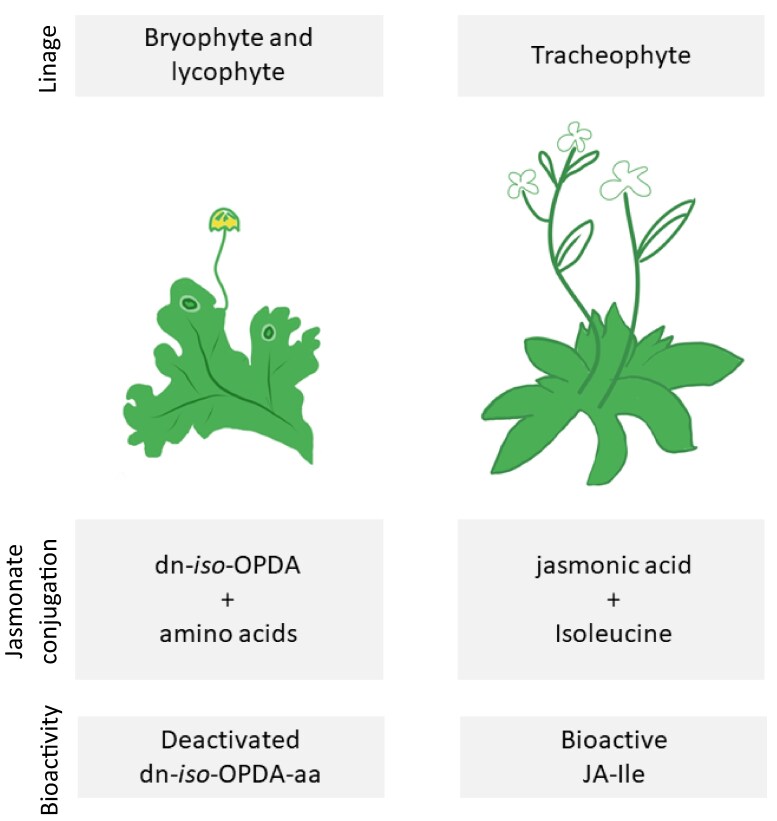
Evolution of different JA signaling pathways in land plant lineages. In bryophytes and lycophytes (represented on the left); JA OPDA’s conjugation to amino acids leads to deactivated form dn-iso-OPDA-aa. In vascular plants (represented on the right); the JA conjugation to Ile leads to bioactive JA-Ile form.

In a recent *Plant Physiology* paper by Liang et al. (2024), the authors used Marchantia to shed light on the metabolites regulated by dn-OPDA. Using untargeted liquid chromatography-mass spectrometry, the authors identified an unknown subset of metabolites—dn-OPDA-amino acid conjugates (dn-OPDA-aas). Subsequent targeted metabolomic profiling identified a significant accumulation of dn-OPDA-aas in response to wounding and herbivory stress. To identify evolutionary conservation of this response, authors looked into the dn-OPDA-aas levels in other species. The same dn-OPDA-aas accumulation in response to stress was found in other lycophyte and bryophyte species, while the accumulation was missing in angiosperms or charophytes, as expected since these species lack dn-OPDA.

Conjugation with amino acids is not an exclusive characteristic of JAs. Other hormones are also conjugated to amino acids through action of the GRETCHEN HAGEN 3 (GH3) enzyme family (Jez 2022). Reasoning that the same enzyme family could be responsible for the conjugation step in Marchantia, authors used CRISPR-Cas9 to generate loss-of-function mutants of Mp*GH3A*. In contrast to the wild-type plants, Mp*gh3a* mutant plants were not able to accumulate dn-iso-OPDA-aa conjugates upon wounding.

Conjugating hormones to amino acids can lead to 2 different responses, resulting in a bioactive or inactive metabolite (Staswick and Tiryaki 2004). To better understand the biological response elicited by dn-iso-OPDA-aa conjugates in Marchantia, authors performed RNA-seq analysis of mutant Mp*gh3a* and wild-type plants. Importantly, they found the Mp*gh3a* mutant plants that are not able to conjugate dn-OPDA-aa and have induced expression of dn-OPDA-related genes, suggesting that the unconjugated form is the active form. A subsequent test of resistance also showed that Mp*gh3a* plants are more resistant to herbivory attack.

In this work, Liang et al. (2024) used an elegant methodology to explore the JA pathway activity in the land plant Marchantia. They found that in Marchantia, conjugation of the activating JA leads to its deactivation, whereas by contrast in Arabidopsis, the JA signaling pathway is activated by the conjugated form of JA, JA-Ile. Employing diverse model organisms to study highly complex hormonal pathways allows identification of evolutionary trends and specialized roles of different metabolites.

Interestingly, conjugation is also known as a regulatory mechanism for other hormones. Hayashi et al. (2021) reported that IAA inactivation by conjugation with amino acids is present in vascular plants (Hayashi et al. 2021). Furthermore, precursor of ethylene, 1-aminocyclopropane-1-carboxylic acid, is regulated by conjugation (Van de Poel and Van Der Straeten 2014). However, the biological role of aminocyclopropane-1-carboxylic acid conjugates has not been well studied. Considering the wide range of processes regulated by phytohormones, balancing the levels of their active form in every moment is a very important developmental and defense checkpoint. The latest work from Liang et al. (2024) is an exciting step in understanding how we can regulate these checkpoints to improve plant performance under attack.
